# A single-cell atlas reveals the heterogeneity of meningeal immunity in a mouse model of Methyl CpG binding protein 2 deficiency

**DOI:** 10.3389/fimmu.2022.1056447

**Published:** 2023-01-10

**Authors:** Huiping Li, Meixin Hu, Zhuxi Huang, Yi Wang, Ying Xu, Jingxin Deng, Ming Zhu, Weijun Feng, Xiu Xu

**Affiliations:** ^1^Department of Child Health Care, Children’s Hospital of Fudan University, National Children’s Medical Center, Shanghai, China; ^2^Institute of Pediatrics, Children’s Hospital of Fudan University, Shanghai, China; ^3^Shanghai Key Laboratory of Medical Epigenetics, International Co-laboratory of Medical Epigenetics and Metabolism, Institutes of Biomedical Sciences, Shanghai Medical College, Fudan University, Shanghai, China

**Keywords:** methyl CpG binding protein 2, Rett syndrome, meningeal immunity, mouse model, central nervous system, single-cell analysis

## Abstract

Methyl CpG binding protein 2 (MeCP2) is a DNA methylation reader protein. Mutations in *MeCP2* are the major cause of Rett syndrome (RTT). Increasing evidence has shown that dysregulated immunity and chronic subclinical inflammation are linked to MeCP2 deficiency and contribute to RTT development and deterioration. The meninges surrounding the central nervous system (CNS) contain a wide repertoire of immune cells that participate in immune surveillance within the CNS and influence various brain functions; however, the characterization and role of meningeal immunity in CNS with MeCP2 deficiency remain poorly addressed. Here, we used single-cell sequencing to profile Mecp2-deficient meningeal immune cells from the dura mater, which has been reported to contain the most meningeal immune cells during homeostasis. Data showed that the meninges of *Mecp2*-null mice contained the same diverse immune cell populations as control mice and showed an up-regulation of immune-related processes. B cell populations were greater in *Mecp2*-null mice than in control mice, and the expression of genes encoding for immunoglobulins was remarkably higher. Mecp2-deficient meninges also contained more cytotoxic CD8^+^ T cells than control meninges. With increased interferon-γ transcription in T and natural killer cells, meningeal macrophages showed decreased suppression and increased activity in Mecp2-deficienct mice. Together, these findings provide novel insights into meningeal immunity, which is a less studied aspect of neuroimmune interactions in *Mecp2*-mutated diseases, and offer an essential resource for comparative analyses and data exploration to better understand the functional role of meningeal immunity in RTT.

## Introduction

Methyl CpG binding protein 2 (MeCP2) is a DNA methylation reader protein, which is able to recognize DNA and histone methylation marks and acts as a methylation-dependent transcriptional modulator within the context of chromatin (1). MeCP2 exerts both transcriptionally repressive and activating functions by interacting with various cofactors, thereby affecting a myriad of genes. *MeCP2* mutations account for 90-95% of classic Rett syndrome (RTT) cases and typically cause the deterioration of acquired psychomotor skills, including the regression of motor and communicative skills, repetitive hand movements, seizures, irregular breathing, ataxia, and autistic features (2).

Although the loss of functional MeCP2 in neurons is thought to cause the majority of symptoms associated with RTT, increasing evidence has shown that dysregulated immunity and chronic subclinical inflammation are also linked to MeCP2 deficiency (3). For instance, several studies have highlighted the role of microglia, which are the primary brain-resident macrophages, in RTT during brain development (4, 5). Additionally, MeCP2 can regulate T cells by influencing the expression of the Forkhead box P3 (Foxp3) transcription factors (6), and MeCP2 deficiency has been associated with enhanced NF-κB signaling in human peripheral blood mononuclear cells (PBMCs) and the human monocyte line THP1 (7).

The meninges surrounding the central nervous system (CNS) comprise a triple layer of membranes, including the pia mater, arachnoid mater, and dura mater. In recent years, the meninges have been found to not only physically protect the CNS, but to also contain a wide repertoire of immune cells that constitute meningeal immunity. Meningeal immune cells and the cytokines they produce participate in immune surveillance within the CNS; influence the response to CNS injury (8) and chronic neurodegenerative conditions; and regulate higher brain functions, such as cognition and social behavior (9). Notably, meningeal macrophages are lost during disease progression in *Mecp2*-null mice (10). Although macrophages are the major cell population in the meninges (11, 12), the characteristics and roles of meningeal immunity in the CNS with MeCP2 deficiency remain poorly understood.

Here, we applied a single-cell sequencing to profile Mecp2-deficient meningeal immune cells from the dura mater, which has been reported to have the most numerous meningeal immune cells in homeostasis (13).

## Methods

### 2.1 Mice

Female B6.129P2(C)-Mecp2^tm1.1Bird^/J (Heterozygous *Mecp2* knockout, Jax:003890) mice were purchased from the Jackson Laboratory (Bar Harbor, ME, USA) and maintained in laboratory on a C57BL/6 background. The mice were maintained and bred in-house under standard 12-h light-dark cycle conditions. They were provided with standard rodent chow and sterilized tap water ad libitum. Male B6.129P2(C)-Mecp2^tm1.1Bird^/J mice (Hemizygote *Mecp2* knockout, *Mecp2*-null mice) at 4-8 weeks of age were used for the experiments, and age-matched wild-type (WT) mice were used as controls. All experiments were approved by the Institutional Animal Care and Use Committee of Fudan University (No. 2019-289).

### 2.2 Isolation of CD45-positive cells from whole dura matter

One-month-old mice were euthanized by an overdose of Avertin (2, 2, 2-Tribromoethanol, 20 mg/mL) and transcardially perfused with 20 mL ice-cold phosphate-buffered saline (PBS). The mice were immediately decapitated posterior to the occipital bone. After the removal of the overlying skin and muscles from the skull, the dorsal part of the skull was carefully removed to isolate the dura. Then, the dura was cut into small pieces in ice-cold RPMI 1640 medium (Gibco, Thermo Fisher Scientific, Waltham, MA, USA) and incubated with an enzyme mix (0.25% Trypsin and 10 U mL^-1^ collagenase type I) at 37°C for 15 min. The solution was gently passed through 70 mm nylon mesh cell strainers using a sterile plastic plunger to yield a single-cell suspension. The single-cell suspension was then centrifuged at 300 g for 10 min. After the supernatant was removed completely, the cell pellet was resuspended in FACS buffer (pH 7,4; 0.1 M PBS; 1 mM Ethylene-Diamine-Tetra-Acetic acid (EDTA); 1% bovine serum albumin (BSA)). CD45-positive cells were subjected to magnetic separation using CD45 MicroBeads (Miltenyi Biotec, Bergisch Gladbach, Germany) according to the manufacturer’s instructions. The cells were placed on ice during all steps except during enzymatic digestion and were prepared for single-cell RNA sequencing (scRNA-seq) or flow cytometry.

### 2.3 ScRNA-seq using 10x Genomics platform

Single CD45^+^ immune cells were freshly isolated from the dura using the procedure described above. For *Mecp2*-null mice, dura from three individual mice were pooled and the experiments were repeated twice. For WT mice, the dura from two and three individual mice were pooled in two repeated experiments. Sequencing was performed by OE Biotech Co., Ltd. (Shanghai, China). Cellular suspensions were loaded onto a GemCode Single Cell Instrument (10x Genomics, Pleasanton, CA, USA) to generate single-cell gel beads-in-emulsion (GEMs) and scRNA-seq libraries using Chromium Next GEM Single Cell 3′ Gel Beads and Library Kit v3.1 (10x Genomics) according to the manufacturer’s instructions. RNA transcripts were uniquely barcoded within single cells and reverse-transcribed into barcoded cDNAs to generate a single multiplexed library. Indexed libraries were sequenced using a NovaSeq 6000 System (Illumina, San Diego, CA, USA) with 2 × 150 paired-end reads.

### 2.4 ScRNA-seq data processing

We processed the unique molecular identifier (UMI) count matrix using the Seurat R package (v3.1.1) (14). Cellular barcodes were demultiplexed using the Cell Ranger software pipeline (v5.0.0) provided by 10× Genomics. Cells with log10GenesPerUMI <0.7 were filtered out to remove low-quality cells and likely multiplet captures. Low-quality cells with >10% of the counts belonging to mitochondrial genes and >5% of the counts belonging to hemoglobin genes were discarded. DoubletFinder package (v2.0.2) was used to identify potential doublets (15). After applying these quality control criteria, 12,129 cells from *Mecp2*-null mice and 13,794 cells from WT mice were included in downstream analyses. Library size normalization was performed using the NormalizeData function in Seurat. Gene expression measurements for each cell were normalized to total expression using “LogNormalize”, multiplied by a scaling factor (10,000 by default), and the results were log-transformed.

Cells were visualized using a two-dimensional Uniform Manifold Approximation and Projection (Umap) algorithm with the RunnUMAP function. The FindVariableGenes, FindClusters, and FindAllMarkers functions in Seurat were used to select variable genes, cluster cells, and identify marker genes, respectively. Differentially expressed genes (DEGs) were identified using the FindMarkers function. P value < 0.05 and |log2foldchange| > 0.58 were set as the thresholds for significant differential expression. Gene ontology (GO) enrichment, Kyoto encyclopedia of genes and genomes (KEGG) pathway enrichment analysis, gene set variation analysis (GSVA), single-cell rEgulatory network inference and clustering (SCENIC), cell-cell communication, and pseudotime analysis were performed as described in previous studies (16–20).

### 2.5 Intracisterna magna injection

**Figure 1:**
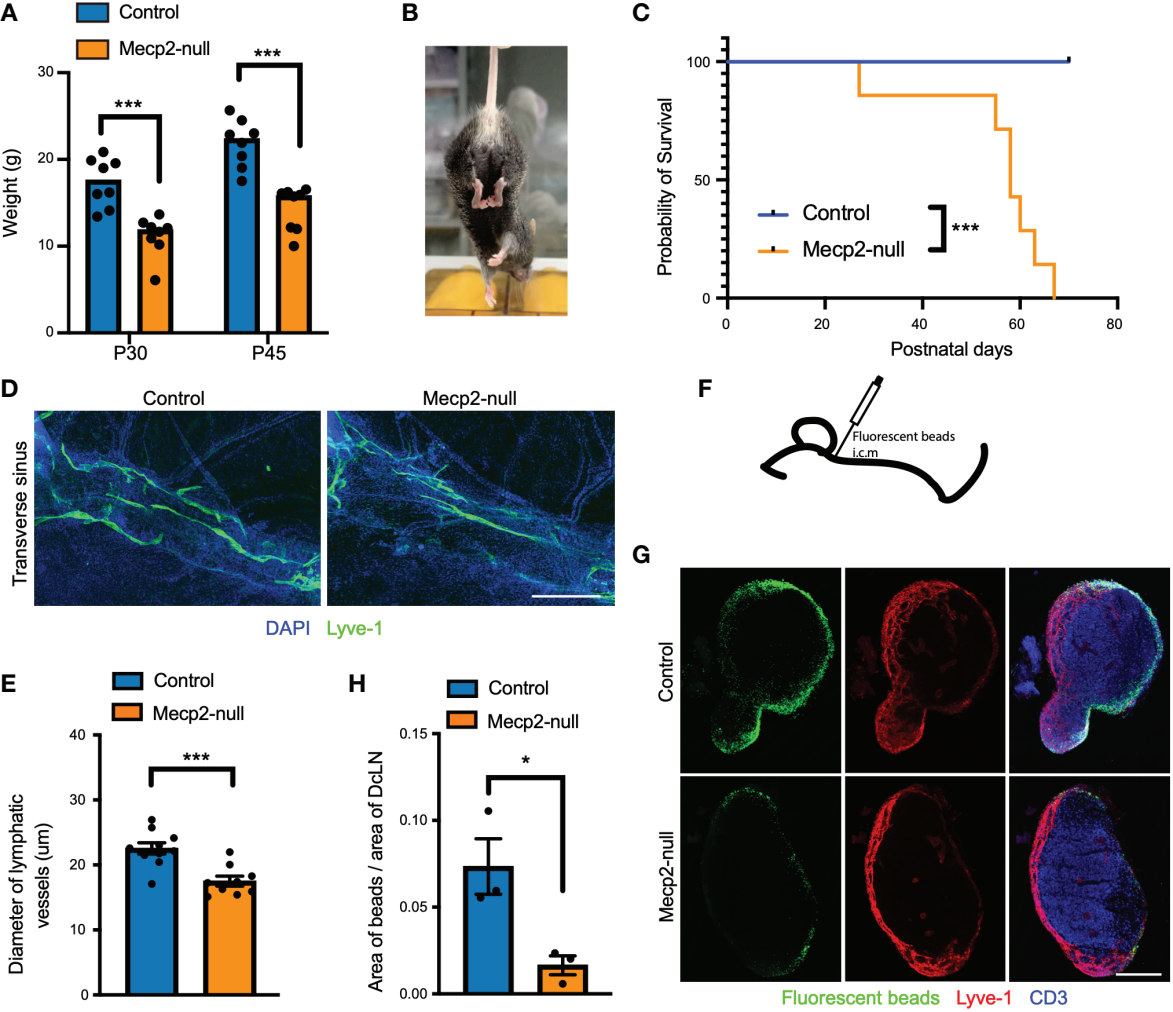
RTT-like phenotypes and deficiency of meningeal lymphatics in *Mecp2*-null mice. **(A)** Bar graph showing retarded growth of *Mecp2*-null mice compared to control at postnatal days 30 and 45 (n=8). *P*=0.0003 (P30) and *P*=0.00006 (P45), multiple t tests. **(B)** Representative images of hindlimb clasping in a *Mecp2*-null mouse at postnatal day 45. **(C)** Kaplan-Meier survival plot for control and *Mecp2*-null mice. (n=7 per group). *P*=0.0001 by the Log-rank test. **(D)** Representative images of the transverse sinus of dura maters with Lyve-1 (green) staining for 1-month-old control and *Mecp2*-null mice. Scale bars: 500μm. **(E)** Quantification of the diameter of Lyve-1^+^ lymphatic vessels (n=10 for control mice; n=9 for *Mecp2*-null mice). Data represent the mean ± SEM. *P* = 0.0005, two-tailed unpaired *t* test. **(F)** Schematic of intracisterna magna injection with fluorescent beads. **(G)** Representative images showing dCLNs with fluorescent beads (green), Lyve-1 (red), and CD3 (blue) staining in control and *Mecp2*-null mice. Scale bars: 250μm. **(H)** Quantification of the percentage of fluorescent beads coverage of the dCLNs from control and *Mecp2*-null mice (n=3 per group). Data represent the mean ± SEM. *P* = 0.0281, two-tailed unpaired *t* test. *p < 0.05, ***p < 0.001.

For meningeal lymphatic drainage experiments, intracisterna magna (i.c.m.) injection was performed as described previously (21). After mice were anaesthetized by intraperitoneal injection with Avertin (250μL/10g), skin on the back of the neck was shaved and cleaned with 75% ethanol, and a 1 cm midline incision was made to expose the cisterna magna. Next, the cisterna magna was injected slowly with 2 μL of fluorescent beads (FluoSpheres Carboxylate-Modified Microspheres, yellow-green, 0.5μm, ThermoFisher) using micro-syringes (**Figure 1F**). After injection, the needle was left in place for 2 min to avoid cerebrospinal fluid (CSF) backflow. The mice were then sutured and allowed to recover on a heating pad. Deep cervical lymph nodes (dCLNs), which are the major meningeal lymphatic draining lymph nodes (21, 22), were harvested 2 h after i.c.m. injection for immunofluorescence staining.

### 2.6 Flow cytometry

CD45-positive cells isolated from the dura mater were stained for extracellular markers using rat anti-CD45 FITC-conjugated (11-0451-82; eBioscience), rat anti-CD3 PE-conjugated (12-0193-82; eBioscience), and rat anti-CD11b APC-conjugated (17-0112-82; eBioscience). Live/dead cells were selected using a Zombie Aqua Fixable Viability Kit (423101, BioLegend). Fluorescence data were collected using a FACSCelesta flow cytometer (BD Company) and analyzed using FlowJo software (v10). Cells were gated using the height, area, forward and side scatter, and live cells with negative Zombie Aqua staining. Cells were gated for the appropriate markers of cell types.

### 2.7 Immunofluorescence staining

After transcardial perfusion with ice-cold PBS and 4% paraformaldehyde (PFA), whole dura maters with the skullcap or dCLNs were dissected from one-month-old mice and post-fixed in 4% PFA at 4 °C. Dura maters were then peeled from the skullcap. The dCLNs were dehydrated using PBS containing 30% sucrose and sliced into 40 μm-thick sections onto gelatin-coated slides. For immunohistochemistry, dura maters or dCLNs sections were incubated in blocking buffer (PBS containing 1% BSA, 2% donkey serum, 0.2% Triton X-100, and 0.1% Tween 20) for 1 h at room temperature (RT). Then, dura maters or sections were moved to appropriate dilutions of the following primary antibodies in blocking buffer for overnight at 4°C: rat anti-CD3 eFluor 660-conjugated (1:200; 50-0032-82; eBioscience), rat anti-Lyve-1 AF488-conjugated (1:200; 53-0443-82; eBioscience), rat anti-Lyve-1 eFluor 570-conjugated (41-0443-82, eBioscience), rat anti-IFN-γ PE/Cyanine7-conjugated (1:200; 505826; BioLegend), and rabbit anti-MeCP2 (1:200; 3456; Cell Signaling). Goat anti-rabbit Alexa Fluor 594 secondary antibody (1:1000; A-11012; Invitrogen) were incubated 1 hour at RT in necessity. Before whole-mounted with Aqua-Mount (Lerner) under coverslips, dura maters or dCLNs sections were staining with 1:10000 DAPI (Sigma-Aldrich) and washed with PBST (PBS containing 0.2% Triton X-100 and 0.1% Tween 20) 3 times for 15 min at RT.

Images were acquired using Leica TSC SP8 confocal system with a 10× (0.4 NA), 20× (0.75 NA), or 40× (0.85 NA) objectives. FIJI software (NIH) was used for image quantitative assessments. The diameter of lymphatic vessels was measured each 50 μm alongside the transverse sinus of dura mater, and the mean was calculated for each sample. Bead coverage in the dCLNs was quantified by dividing the area of GFP fluorescence over the area of the lymph node, as indicated by Lyve-1 and CD3 staining. The density of CD3-positive cells or IFN-γ was determined by dividing the number of CD3-labeled cells or the IFN-γ-positive area per section by the area of dura maters.

### 2.8 Statistical analysis

Statistical analysis was performed by GraphPad PRISM 8. Data were compared using two-tailed unpaired Student’s *t*-tests, multiple t tests, and Kaplan-Meier survival plot. Statistical significance was defined as p < 0.05.

## Results

### 3.1 RTT-like phenotypes and meningeal lymphatics in *Mecp2*-null mice

As reported previously (5, 23), *Mecp2*-null mice (male B6.129P2(C)-Mecp2^tm1.1Bird^/J) displayed RTT-like phenotypes, including significantly retarded growth, hindlimb clasping, and short postnatal life expectancy (**Figures 1A–C**). In addition, we observed meningeal lymphatics deficiency in *Mecp2*-null mice, as indicated by a decrease in the diameter of meningeal lymphatic vessels (**Figures 1D, E**) and a decrease in the drainage of CSF fluorescent beads from the cisterna magna into dCLNs (**Figures 1G, H**). Meningeal lymphatics play important roles in the development and recirculation of CNS immune cells (21, 24); hence, these observations suggest that meningeal immunity may be altered in *Mecp2*-null mice.

### 3.2 Meningeal immune cell diversity

**Figure 2:**
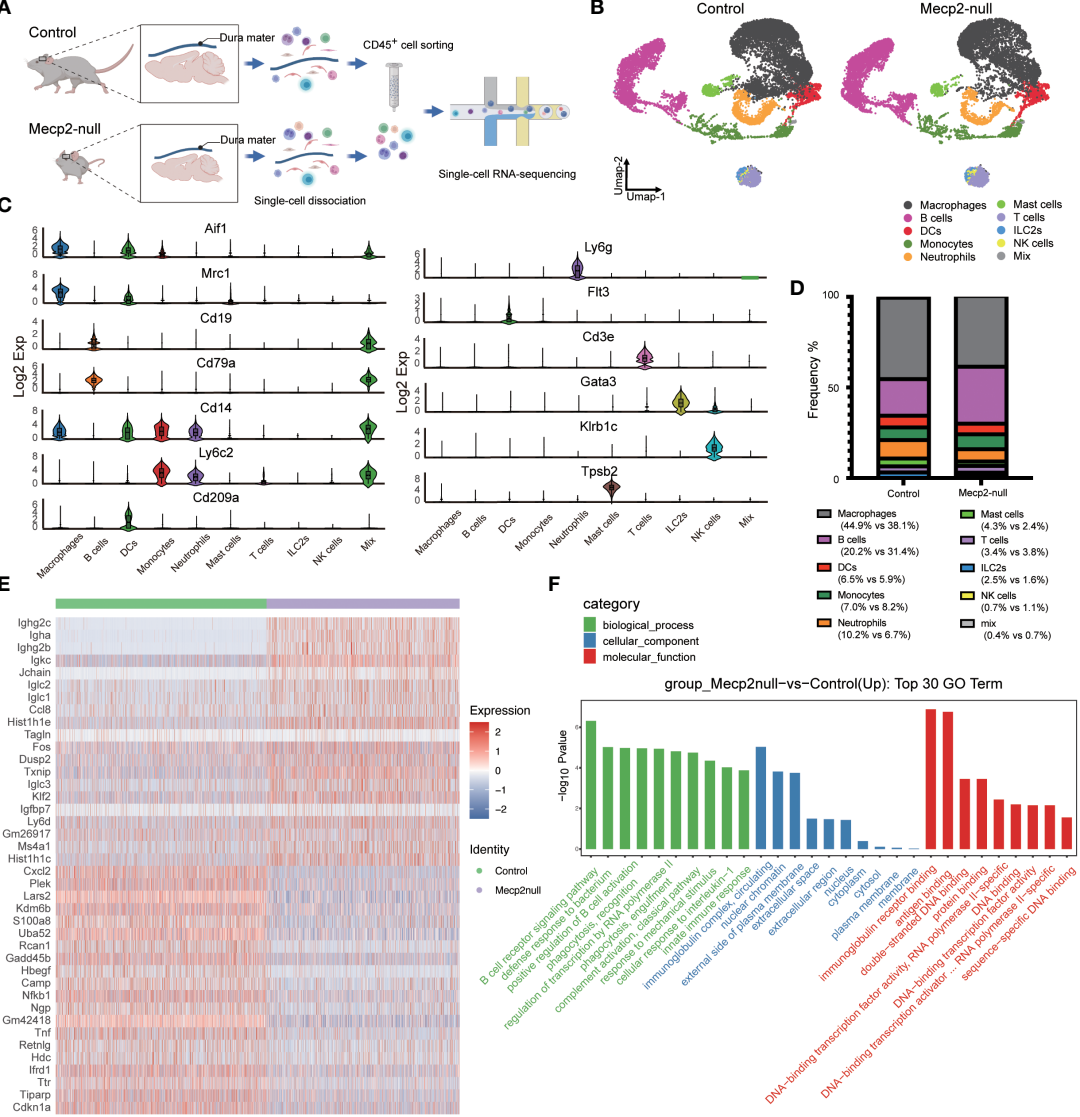
Single-cell transcriptomics survey of dura immune cells from WT and Mecp2-deficienct mice. **(A)** Schematic of the experimental approach (created with Biorender.com). 10× chromium scRNA-seq for CD45^+^ immune cells isolated from dura maters of 1-month-old WT (control) and Mecp2-deficienct (*Mecp2*-null) mice. **(B)** Umap plot displaying 10 dura immune cell types from control and *Mecp2*-null mice. Colored show annotated cell types. **(C)** Violin plots showing the expression levels of 13 specific marker genes for different cell types. **(D)** Frequency of each cell type in control and *Mecp2*-null mice. **(E)** Heatmap of group-specific DEGs. Color indicates relative gene expression, with representative genes shown together. **(F)** The top 30 most enriched GO terms for DEGs in control and *Mecp2*-null group.

To identify meningeal immune changes associated with Mecp2 deficiency, we generated 12,129 and 13,794 high-quality single-cell transcriptomes for meningeal immune cells (CD45^+^ cells) from six *Mecp2*-null and five WT mice, respectively (**Figure 2A**). Cells from the various mice were also pooled into a single dataset, and color-coded Umap plots were generated to visualize the origin of each cell (**Figure 2B**). Immune cells formed 18 major clusters that expressed varying levels of *Ptprc*, a gene encoding CD45 (**Supplementary Figure S1A, B**). We classified the different cell types based on the expression levels of the most variable genes and the shared expression features of known marker genes (**Figure 2C**; **Supplementary Figure S1C**). Consistently with the results of previous studies on Mecp2 in immune system (6, 10), we found that meningeal immune cells expressed Mecp2 (**Supplementary Figure S2**).

The dura maters of both control and *Mecp2*-null mice contained diverse immune cell populations, including macrophages, B cells, dendritic cells (DCs), monocytes, neutrophils, mast cells, T cells, innate lymphoid cells-2 (ILC-2), and natural killer (NK) cells (**Figure 2B**). Obvious differences in the cell populations were observed between *Mecp2*-null mice and control mice, with a loss of macrophages and an increase in B cells (**Figure 2D**). According to previous studies (5, 10), Mecp2 deficiency causes the decline of macrophages and microglia, which are specific types of myeloid cells resident in the brain. Surprisingly, we found that the frequency of B cell populations was almost 1.5 times higher in *Mecp2*-null mice. The expression of genes encoding immunoglobulins, such as *Ighg2c, Igha, Ighg2b, Igkc, Iglc2, Iglc1*, and *Iglc3*, was also markedly higher in *Mecp2*-null mice than in control mice (**Figure 2E**). Other DEGs included CC chemokine (*Ccl8*), histones (*Hist1h1e, Hist1h1c*), transcription factor (*Fos*), protein phosphatase (*Dusp2*), and thioredoxin-binding protein (Txnip) (**Figure 2E**; **Supplementary Data Sheet 1**). GO enrichment analysis revealed the up-regulation of immune-related processes and regulation of transcription in *Mecp2*-null mice, including the B cell receptor signaling pathway, innate immune response, and DNA-transcription factor activity (**Figure 2F**).

### 3.3 Meningeal B cell activity

**Figure 3:**
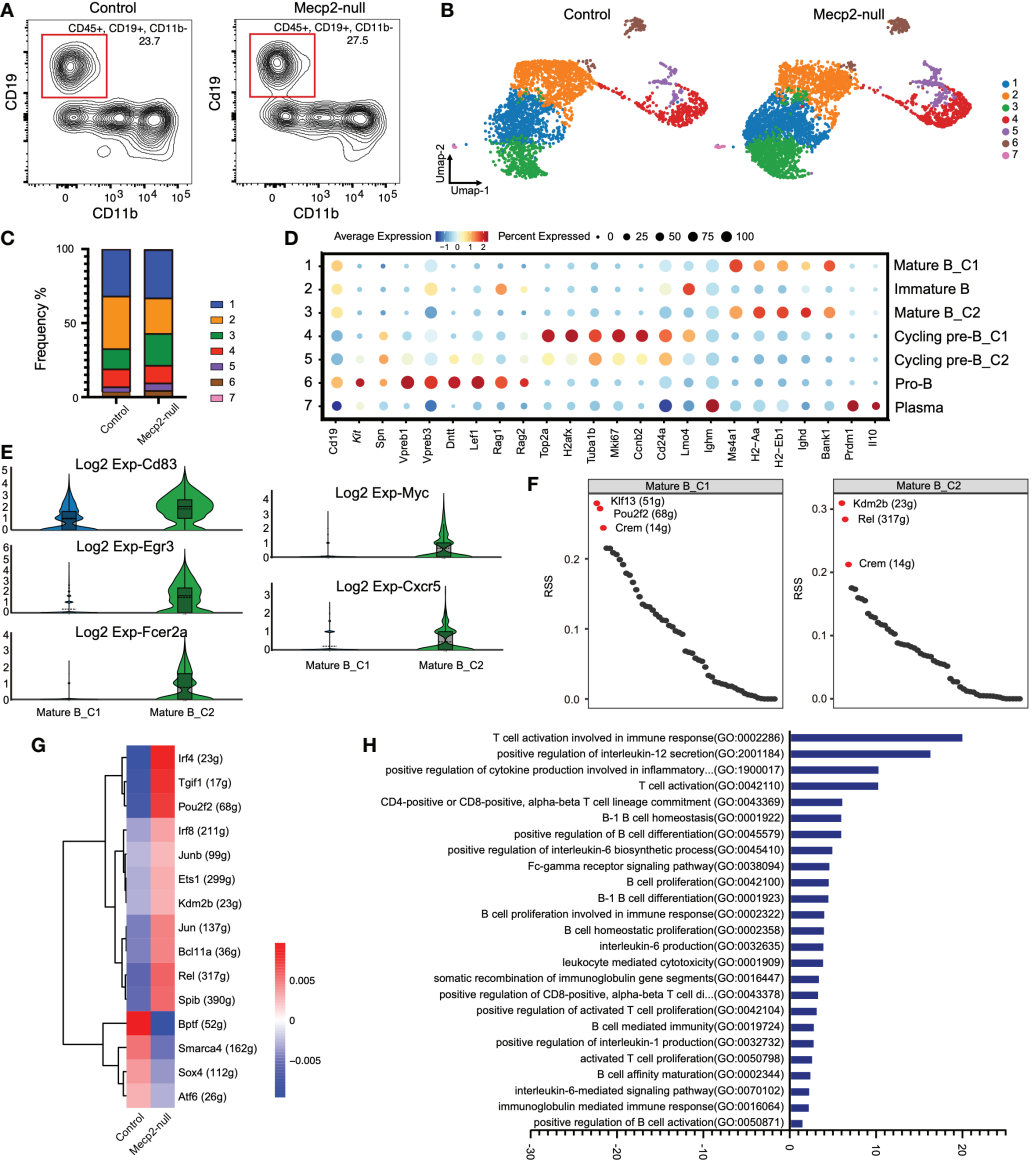
Gene expression and characterization of B cells compared to control and *Mecp2*-null mice. **(A)** Representative flow cytometry contour plot showing the proportion of B cells within the CD45^+^ population in the dura of control and *Mecp2*-null mice. B cells were gated as CD45^+^CD19^+^ and CD11b^-^. n=3 for each group. **(B)** Distribution of seven B cell clusters on the Umap plot for two groups. Clusters are rank-ordered by size. **(C)** Bar plots showing cell cluster distribution within different groups. **(D)** Dot plots displaying the relative expression of selected B cell marker genes (columns) across clusters (rows) in **(B)**. Dot size indicates the fraction of cells in the clusters that express a gene. Color indicates the Z-score of mean gene expression in the cluster (Z-score of average log2). **(E)** Violin plots of *Cd83, Egr3, Fcer2a, Myc*, and *Cxcr5* expression in control and *Mecp2*-null mice. **(F)** Rank for regulons in cluster1 and cluster2 of mature B cells (Mature B_C1 and Mature B_C2) based on regulon specificity score (RSS). **(G)** Heatmap of regulon activity score (RAS) in two groups. Rows represent different regulons and columns represent mouse groups. **(H)** Bar plot showing different enriched pathways comparing in *Mecp2*-null and control mice determined using GSVA. *T*-values > 0 represent up-regulated pathways.

Although B cells accounted for the main changes observed in the meninges of *Mecp2*-null mice, little is known about their characteristics under homeostasis. We also observed an increase in the B cell population in the dura maters of *Mecp2*-null mice by flow cytometry (**Figure 3A**). The B cell population was then re-clustered into seven clusters (C1 to C7) on a single Umap space (**Figure 3B**). B cells from the *Mecp2*-null and control mice occupied overlapping territories; however, the frequencies of C2 and C3 were reversed between the two groups (**Figure 3C**). Consistent with previous studies by Wang et al. (25) and Brioschi et al. (26), the seven sub-clusters formed a consecutive development trajectory of B lineage cells (**Figure 3D**). Mature B cells (C1 and C3) expressed high levels of *Ighd, Ighm*, and *Ms4a1*. Meanwhile, immature B cells (C2) expressed high levels of *Ighm*, but not *Ighd*. In cycling pre-B cells (C4 and C5) the expression of *Top2a, H2afx*, and *Mki67* was upregulated. Pre-B cells (C6) expressed high levels of *Vpreb1, Vpreb3, Dntt, Lef1*, and *Rag1*, while plasma cells (C7) highly expressed *Prdm1* and *Il10*.

In *Mecp2*-null mice, the C3 cluster of mature B cells (Mature B_C2) expanded and strongly expressed B cell activation markers, such as *Cd83, Egr3, Fcer2a, Myc*, and *Cxcr5* (**Figure 3E**). Analysis of the regulon most highly associated with this subset revealed a high regulon specificity score for transcription factors *Kdm2b, Rel*, and *Crem* compared to the mature B_C1 cluster (C1) (**Figure 3F**). The regulon activity heatmap also showed increased activities for *Kdm2b* and *Rel* in *Mecp2*-null mice (**Figure 3G**).

In addition, we ranked the estimated pathway activities for individual B cells using GSVA. This approach revealed the strong enrichment of T cell activation involved in the immune response, positive regulation of Il-12 secretion, positive regulation of cytokine production involved in the inflammatory response, and B cell activation and proliferation (**Figure 3H**).

### 3.4 Cytotoxic activity of T lymphocytes

**Figure 4:**
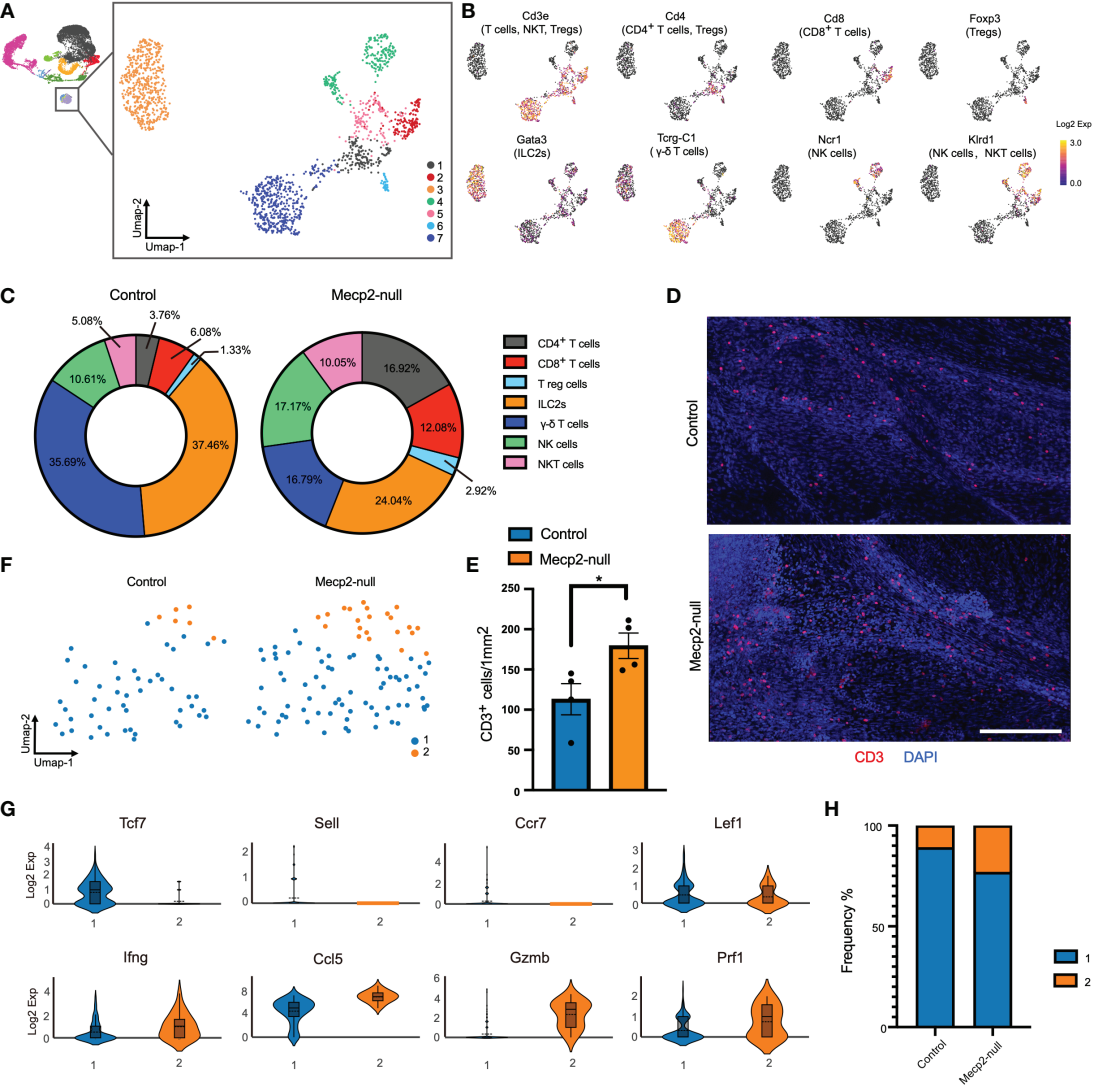
Increased T cell cytotoxicity in Mecp2-deficienct mice. **(A)** Umap plot showing the distributions of seven clusters for T cells, NK cells, and ILC2s. **(B)** Umap plots showing the expression of signature genes for CD4^+^ T, CD8^+^ T, Tregs, γ-δ T, ILC2s, NK, and NKT cells. **(C)** Percentages of each cell population among the total meningeal T cells, NK cells, and ILC2s in control and *Mecp2*-null mice. **(D)** Representative confocal images of CD3 (red)-positive cells in the dura maters of control and *Mecp2*-null mice. Scale bars: 250μm. **(E)** Quantification of the density of CD3-positive cells in the dura maters of control and *Mecp2*-null mice (n=4 per group). Data represent the mean ± SEM. *p < 0.05, *P* = 0.0371, two-tailed unpaired *t* test. **(F)** Umap plots indicating the two sub-clusters of CD8^+^ T cells. **(G)** Violin plots representing the expression of resting state (top row) and cytotoxicity/activated (bottom row) related markers. **(H)** Frequency of two sub-clusters of CD8^+^ T cells in control and *Mecp2*-null mice.

To better understand T cell activation in Mecp2-deficient mice, we re-clustered ILC2s, NK cells, and T cells into seven clusters (**Figure 4A**). The major cell types were identified by defining genes for CD4^+^ T cells (C1) characterized by *Cd3e* and *Cd4* expression; CD8^+^ T cells (C2) characterized by *Cd3e* and *Cd8* expression; ILC2s (C3) characterized by *Gata3* expression; NK cells (C4) characterized by *Nrc1* and *Klrd1* expression and without Cd3e expression; NKT cells (C5) characterized by *Cd3* and *Klrd1* expression; regulatory T cells (Treg, C6) characterized by *Cd3e, Cd4*, and *Foxp3* expression; and γ-δ T cells (C7) characterized by *Cd3e* and *Tcrg-C1* expression (**Figure 4B**). The frequencies of ILC2, NK cell, and T cell populations were affected by Mecp2 deficiency. *Mecp2*-null mice had higher frequencies of CD4^+^ T cells, CD8^+^ T cells, NK, and NKT cells. In contrast, the frequencies of ILC2s and γ-δ T cells were lower (**Figure 4C**). Immunofluorescence staining for CD3 also showed a higher density of CD3 positive cells in dura mater of *Mecp2*-null mice (**Figures 4D, E**).

CD8^+^ T cells are a critical population of MHC I-restricted T cells that directly kill infected or damaged cells. Differential gene expression from the scRNA-seq data of CD8^+^ T cells resolved the subsets and functional states into two subsets (**Figure 4F**). Subset 1 showed higher *Tcf7*, *Sell, CCr7*, and *Lef1* expression, which corresponded with the resting state of CD8^+^ T cells (**Figure 4G**). Subset 2 showed increased expressions of *Ifng* (encoding interferon-γ; IFN-γ), *Ccl5, Gzmb*, and *Prf1*, which are associated with cytotoxicity and activation (**Figure 4H**). The distribution of subset 2 was more enriched in *Mecp2*-null mice than in the control group, suggesting that the cytotoxic activity of T lymphocytes was enhanced in *Mecp2*-null mice.

### 3.5 IFN-γ transcription and cell-cell interactions

**Figure 5:**
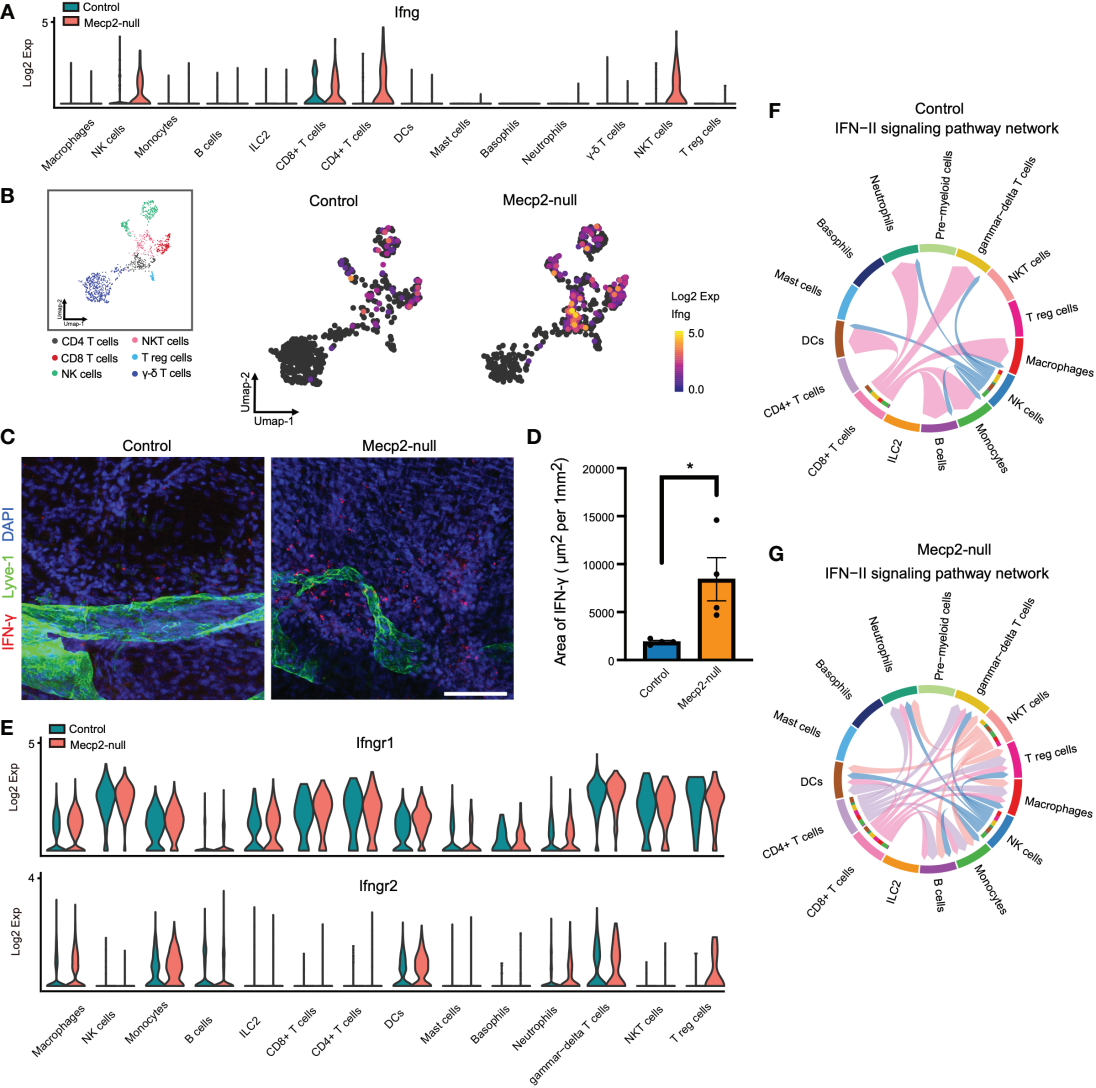
scRNA-seq analysis of IFN-II signaling pathway upregulation in multiple Mecp2-deficient cell populations. **(A)** Violin plots showing Ifng expression in different meningeal immune cells from control and *Mecp2*-null mice. **(B)** Umap plots depicting Ifng expression in the main producer cells. **(C)** Representative confocal images of IFN-γ (red) with lymphatic vessel endothelial receptor 1 (Lyve-1, Green) in the dura maters of control and *Mecp2*-null mice. Scale bars: 100μm. **(D)** Quantification of the area of IFN-γ in the dura maters of control and *Mecp2*-null mice (n=4 per group). Data represent the mean ± SEM. *p < 0.05, *P* = 0.0278, two-tailed unpaired *t* test. **(E)** Violin plots showing the expression of IFN-γ receptors (Ifngr1 and Ifngr2) in different meningeal immune cells in control and *Mecp2*-null mice. **(F)** Chord diagram visualizing cell-cell communication for the IFN-II signaling pathway in WT mice. **(G)** Chord diagram visualizing the cell-cell communication for IFN-II signaling pathway in *Mecp2*-null mice.

CD8^+^ T cells of *Mecp2*-null mice displayed increased expression of *Ifng*, which encodes IFN-γ. IFN-γ is the only member of the type II class of interferons and plays a critical role in both innate and adaptive immunity (27). Yang et al. reported that Mecp2 overexpression results in defective IFN-γ secretion and suggested that MeCP2 could be a regulatory factor in *Ifng* transcription (28). Here, compared to that in control mice, we found that *Mecp2*-null mice had higher *Ifng* expression in NK, CD8^+^ T, CD4^+^ T, and NKT cells (**Figures 5A, B**). Immunofluorescence staining further revealed that IFN-γ expression was higher in the dura maters of *Mecp2*-null mice (**Figures 5C, D**). However, the expression of the IFN-γ receptors, *Ifngr1* and *Ifngr2*, was not significantly affected by Mecp2 deficiency, with the exception of high *Ifngr2* expression in Treg cells (**Figure 5E**). CellChat was used to explore the differences in intercellular communication networks of the IFN-II signaling pathway between control and *Mecp2*-null mice, which were visualized using circle plots. *Mecp2*-null mice showed more complex intercellular communication in the IFN-II signaling pathway (**Figures 5F, G**).

### 3.6 Macrophages activated in response to IFN-γ in *Mecp2*-null mice

**Figure 6:**
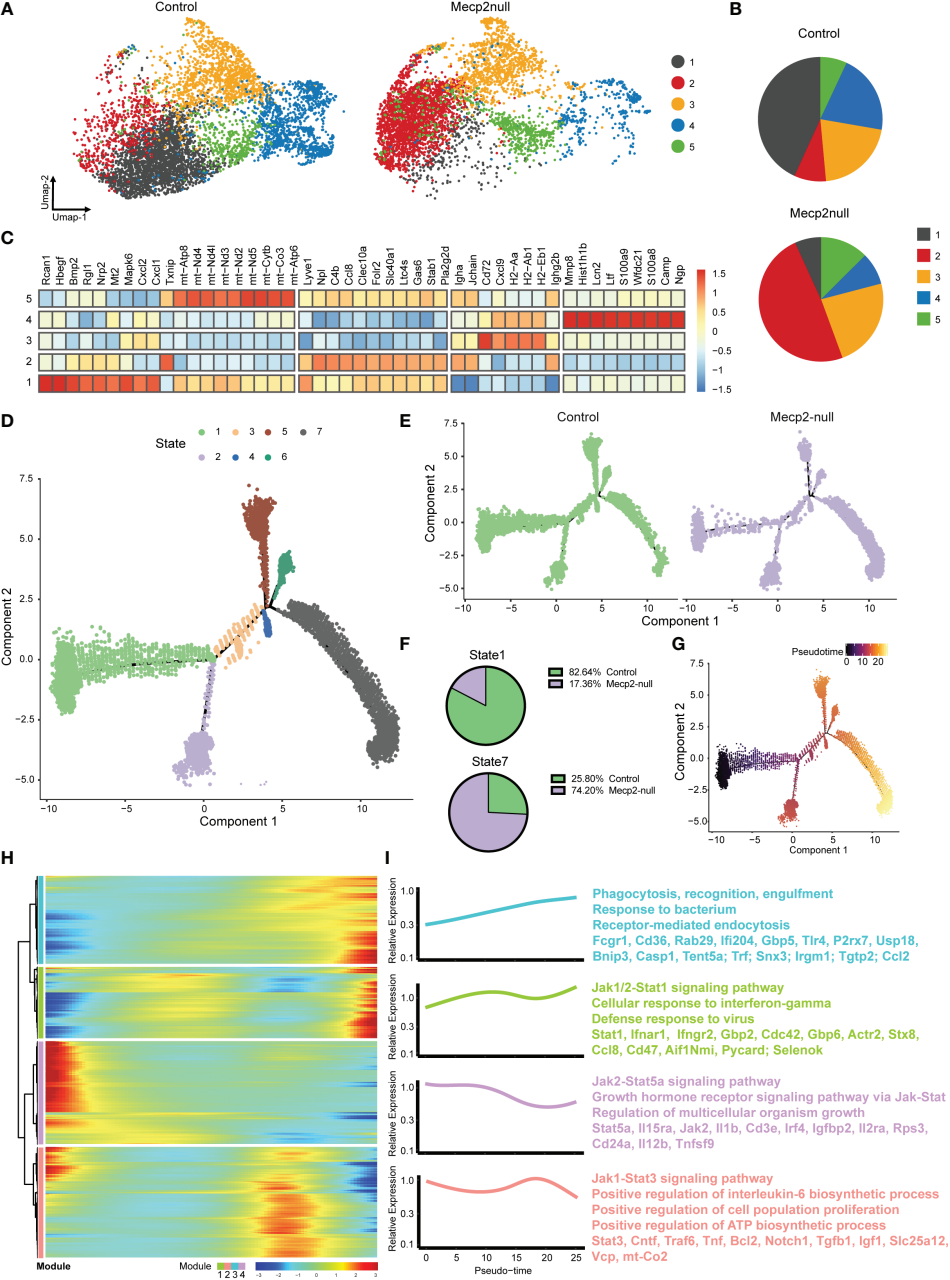
MeCP2 deficiency macrophages have increased response to IFN-γ. **(A)** Umap plots showing the clusters of WT and Mecp2-deficienct macrophages. **(B)** Pie chart showing the distribution of five clusters in WT and Mecp2-deficienct meningeal macrophages. **(C)** Heat map displaying the relative expression fold change (log2) of the top 50 up-regulated genes in macrophage clusters identified in **(A)**. **(D)** Visualization of the seven cell states predicted using the Monocle2 algorithm. **(E)** Trajectories for control and *Mecp2*-null mice. **(F)** Numbers expressed as percentages of control (green) and *Mecp2*-null (purple) macrophages in state 1 (top) and state 7 (bottom) cell populations, respectively. **(G)** Pseudotime of macrophages indicated by gradient color intensity from dark to bright, indicating the progression from the early to late pseudotime. **(H)** Heatmap displaying genes ordering during pseudotime. Color gradient from blue to red indicates low to high relative expression levels. **(I)** The GO and KEGG of enriched functions and pathways for the significantly differentially expressed (SDE) genes.

IFN-γ plays a key role in macrophage activation and reinforces the M1 phenotype (29). To stratify the activity of macrophages, we re-clustered the macrophage population into five subpopulations based on the expression level of signature genes (**Figure 6A**). Although Mecp2-deficient meningeal macrophages mainly overlapped with those in the control group, some obvious differences were observed, especially in subclusters 1 and 2 (**Figure 6B**). We examined the top 50 up-regulated genes in the macrophage subclusters. Subclusters 1, 2, and 5 fell into the MHC II low group (11), which exhibited high *Lyve1* and *Gas6* expression (**Figure 6C**), while subclusters 3 and 4 fell into the MHC II high group, which exhibited high *H2-Aa* and *Cd72* expression (**Figure 6C**). Subcluster 1 displayed increased expression of *Rcan1*, which encodes a small protein that inhibits calcineurin phosphatase activity and acts as a central negative regulator of inflammation (30).

Finally, we performed SCORPIUS trajectory inference on the macrophages. The macrophages were divided into seven states (**Figure 6D**). The cell trajectory patterns of Mecp2-deficient mice shifted from state 1 to state 7 (**Figure 6E**), with gene expression profiles changing dynamically along the trajectory. The most predictive genes were clustered into modules, which revealed the transcriptional gradients of genes that were lost or gained in pseudotime (**Figure 6H**). IFN-γ mediates the polarization of macrophages to an “M1-like” state by activating Janus kinase (Jak)-signal transducer and activator of transcription 1 (Stat1) (31). GO analyses indicated that the late pseudotime, which consisted mainly of state 7, was enriched for the genes involved in phagocytosis, recognition, engulfment, and the Jak1/2-Stat1 signaling pathway. Conversely, early pseudotime mainly consisted of state 1 and was enriched for genes in the Jak2-Stat5a and Jak1-Stat3 signaling pathways (**Figure 6I**). Taken together, these results indicate that macrophages are activated in response to IFN-γ in *Mecp2*-null mice.

## Discussion

Due to the unique position and structural composition of the meninges, and the functional characteristics of meningeal immune cells, meningeal immunity plays an important role in maintaining the CNS under healthy and disease conditions (12, 32). MeCP2 is expressed in immune cells. Polymorphisms in MeCP2 have been linked to increased susceptibility to autoimmune diseases, and alterations in MeCP2 expression levels affect immune function and cytokine production (3, 33). However, little is known about changes in meningeal immunity in the absence of MeCP2 and the role of meningeal immunity in RTT progression. Using single-cell genomic analysis, we explored the meningeal immune niche of pre-phenotypic Mecp2-deficienct mice, which displayed mild symptoms but were less affected by systemic damage. The meninges of *Mecp2*-null mice contained diverse immune cell populations like control mice and showed an up-regulation of immune-related processes, including the B cell receptor signaling pathway, cytotoxic activities of T lymphocytes, and macrophage activities. Furthermore, Mecp2 deficiency increased IFN-γ transcription and cell-cell interactions mediated by the IFN-II signaling pathway.

Interestingly, our findings on meningeal immunity with Mecp2 deficiency have some similarities and differences compared to previous studies. Papini et al. (34) demonstrated that patients with RTT showed a consistent and highly significant increase in the serum IgM fraction and we found that the expression of immunoglobulin-family genes in Mecp2 deficient meningeal immune cells (*Ighg2c, Igha, Ighg2b, Igkc*, etc.) was abnormally high (**Figure 1**). Li et al. (6) found that Mecp2 is essential for maintaining stable Foxp3 expression and the lineage identity of mature Tregs during inflammation, but is dispensable for the initial induction of Foxp3 expression during Treg development. Consistently, we observed no loss in Foxp3 expression or decrease in the number of Tregs in our immune cell population with no inflammatory challenge (**Figure 3B**). Leoncini et al. (35) showed that significantly reduced plasma levels of IFN-γ in patients with *MeCP2*-mutated RTT; however, we observed enhanced IFN-γ transcription in MeCP2-deficient meningeal immune cells (**Figure 4**), indicating that meningeal immunity is unique.

IFN-γ is a dimerized soluble cytokine that was originally identified as a ‘macrophage-activating factor’ (31). IFN-γ concentrations in the brain are elevated in certain pathologies, including multiple sclerosis, cerebral ischemia, and neurotrauma (36). In this study, we found that *Mecp2*-null mice lost a population of meningeal macrophages with high Rcan1 expression, which suppresses macrophage activity and negatively regulates inflammation. SCORPIUS trajectory inference showed that the state enriched for phagocytosis, recognition, engulfment, and the Jak1/2-Stat1 signaling pathway (activated by the IFN-γ receptor) was enhanced in Mecp2-deficienct meningeal macrophages. Thus, the abnormally enhanced innate immune response observed in Mecp2-deficienct meningeal immunity may participate in the onset and progression of RTT by causing low-grade neuroinflammation, as reported by Pecorlli et al. (3). However, the cellular targets of IFN-γ extend beyond immune cells. Due to the exclusion system of cerebrospinal fluid, meningeal soluble mediators, such as cytokines, have limited ability to enter and affect the brain parenchyma under steady-state conditions (37). With the expression of IFN-γ receptors in the membranes of neurons (36), IFN-γ has been known to regulate dendrite morphology, inhibit the formation of excitatory synapses in sympathetic neurons and the neocortex (38), and promote inhibitory currents in GABAergic neurons in the prefrontal cortex (39). Furthermore, studies have shown that reduced dendritic complexity, synaptic strength, and dysfunction of GABAergic neurons contribute towards RTT (40–42).

**Figure 7:**
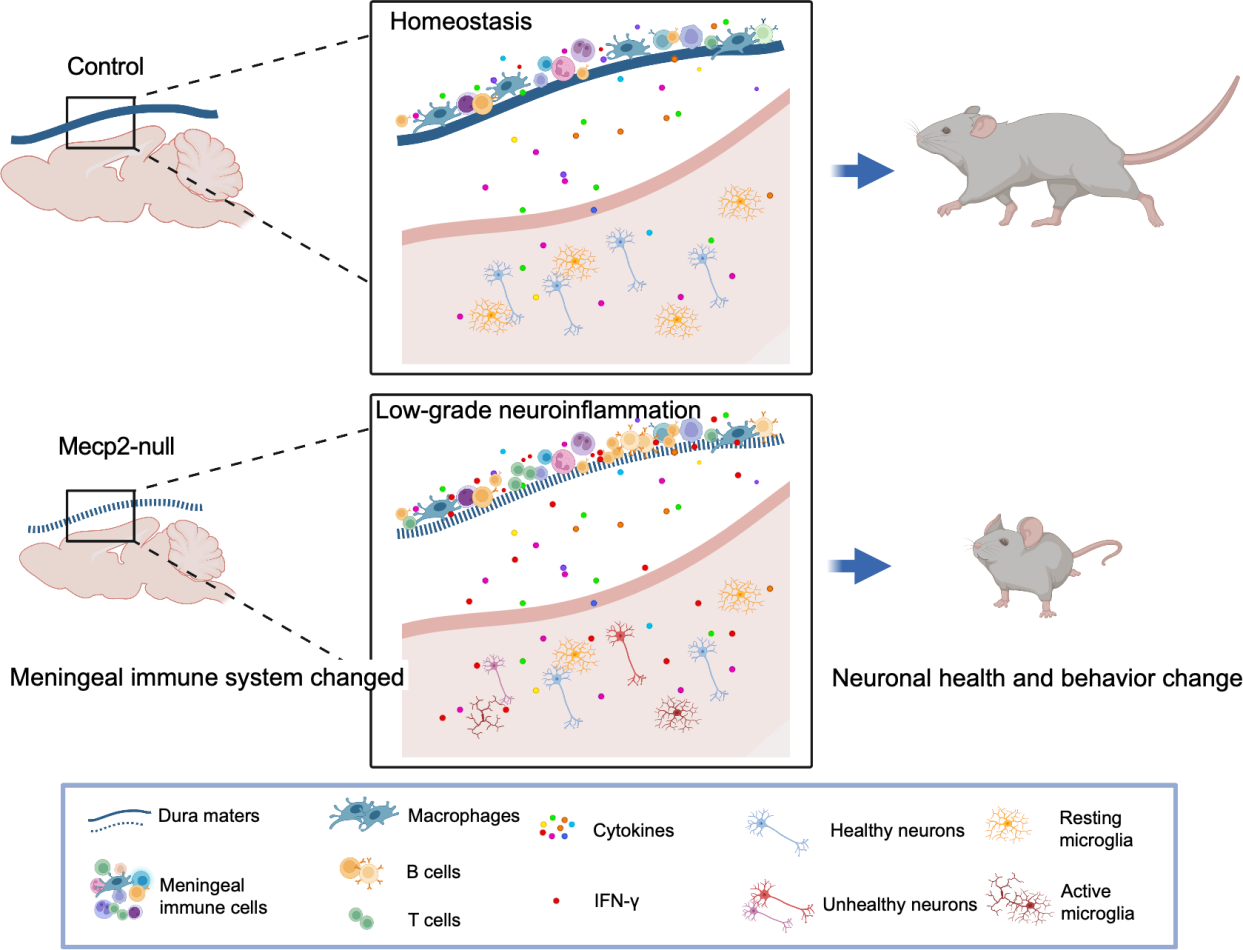
Model for the mechanisms of meningeal immunity in *Mecp2*-null mouse. Illustration of the hypothesis for the mechanisms of meningeal immunity with the progression of disease in *Mecp2*-null mice (created with Biorender.com). Meninges surrounding the brain have functional lymphatic system and abundant of immune cells which play important roles for maintaining the central nervous system in health. *Mecp2*-null mice show deficiency of meningeal lymphatics and activity status altered of immune cells in dura mater. The non-infectious inflammatory response can spread to the CNS parenchyma, causing low-grade neuroinflammation, and further impairing neuronal health and behavior change.

There is still much to understand regarding how meningeal immunity affects neuronal function. It remains unclear how the association of meningeal immunity changes with the progression of *MeCP2-*mutated diseases. Accumulating evidence has shown that meningeal immunity supports the brain function and may play potential roles in neurodegenerative diseases (9, 43, 44). Indeed, *Mecp2*-mutant rodents and humans have severely impaired brain function and progressive aggravation (45). Meningeal immunity is thought to support the brain function by secreting cytokines that could be carried by the CSF to corresponding receptors expressed in the brain parenchyma (32). Deficiency in meningeal-derived cytokines, such as IL-4, IL-13, and IL-17α, has been shown to impaired learning, memory, social, and anxiety-like behaviors (9, 43, 46). Based on our findings, we propose a model associating heterogeneity in meningeal immunity with the progression of disease in *Mecp2*-null mice (**Figure 7**). In *Mecp2*-null mice, immune-related processes including the B cell receptor signaling pathway, cytotoxic T lymphocyte activities, and macrophage activities are up-regulated. This heterogeneity of meningeal immune cells and activity status alters the secretion of cytokines (especially IFN-γ), which can enter the brain parenchyma through CSF and affect neuronal function. However, further research is required to confirm the role of meningeal immunity in RTT development.

In summary, our study provides novel insights into the pathology of *MeCP2-*mutated diseases. The meninges, which contain a wide repertoire of immune cells and participate in immune surveillance in the CNS, showed decreased suppression and increased activity for both innate and adaptive immunocytes in MeCP2-deficienct mice. Furthermore, we found that the overexpression of meningeal IFN-γ might not only modulate the status of immune cells in the meninges, but also affect brain function and contribute towards RTT development and deterioration.

## Data availability statement

The datasets presented in this study can be found in online repositories. The name of the repository and accession number can be found below: NCBI Gene Expression Omnibus; GSE221361.

## Ethics statement

The animal study was reviewed and approved by Institutional Animal Care and Use Committee of Fudan University.

## Author contributions

Conceptualization, HL. Mouse genotyping, MH, ZH, YX, MZ. Meningeal dissection and dissociation, HL, MH, YW, JD. Data analysis, HL, MH. Investigation, HL, WF, and XX. Data curation, MZ. Writing-original draft, HL. Writing-review and editing, WF, and XX. Supervision, WF, and XX. All authors contributed to the article and approved the submitted version.

## Supplementary material

Supplementary Figure 1 Single-cell characterization of meningeal immune cells with typical signature marker genes. **(A)** Umap plot displays distributions of 18 major clusters of meningeal immune cells. **(B)** Log2 expression of *Ptprc* in individual cells on the Umap graph of **(A)**. **(C)** Violin plots showing the expression of signature genes for different immune cell types.

Supplementary Figure 2 Meningeal immune cells express Mecp2. **(A)** Representative confocal images of Mecp2 (red) and Lyve-1(green) staining in the dura mater of 1-month-old WT mice. Scale bars: 50μm. **(B)** Violin plots representing the Log2 expression of *Mecp2* in different meningeal immune cells from WT mice.
