# Supplementary figures and images for: A single-cell atlas reveals the heterogeneity of meningeal immunity in a mouse model of Methyl CpG binding protein 2 deficiency
Source: Front Immunol. 2023 Jan 10;13:1056447. doi: 10.3389/fimmu.2022.1056447 (PMC9871622; doi:10.3389/fimmu.2022.1056447)

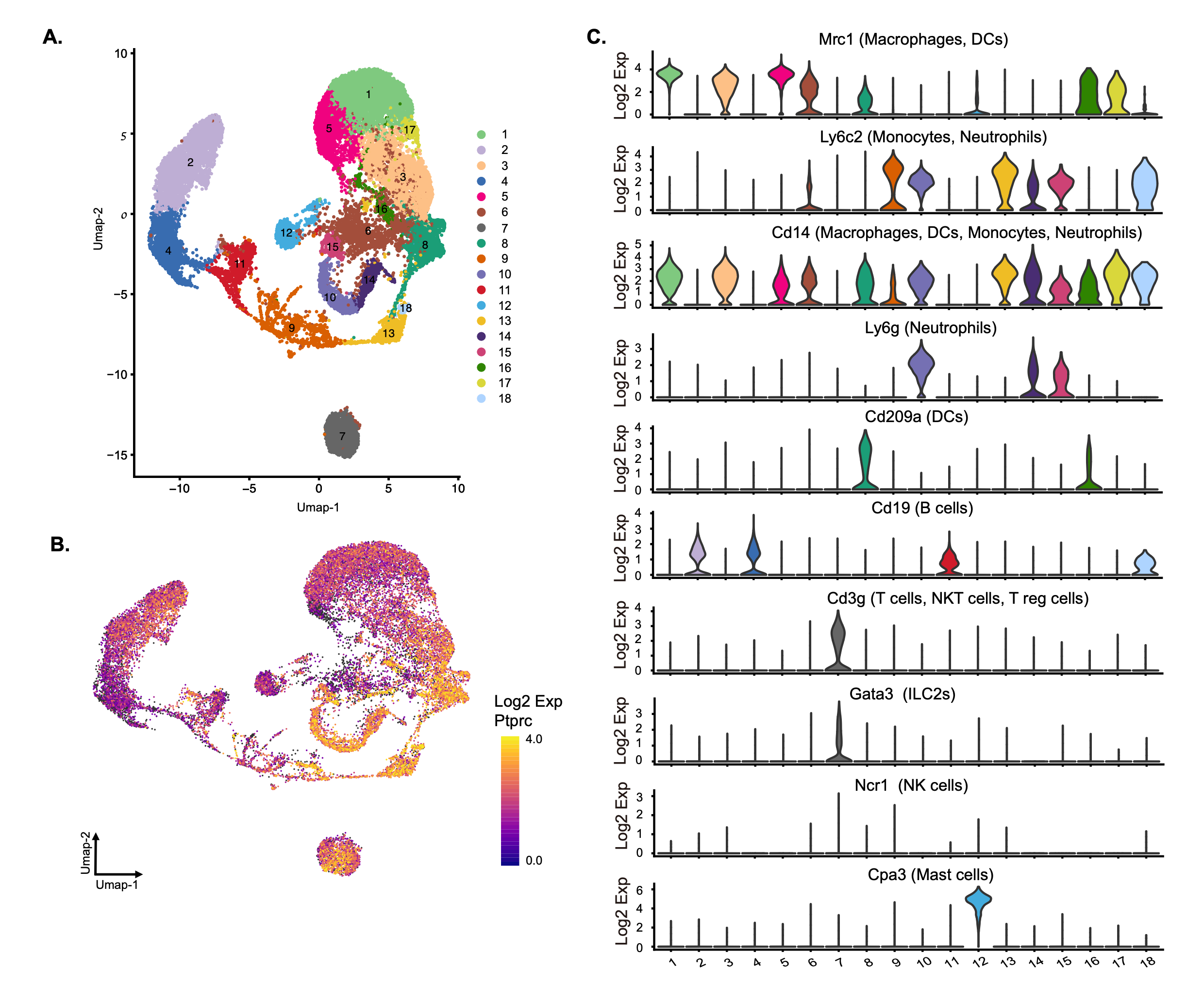

Supplement: Supplementary Figure 1 — Single-cell characterization of meningeal immune cells with typical signature marker genes. (A) Umap plot displays distributions of 18 major clusters of meningeal immune cells. (B) Log2 expression of Ptprc in individual cells on the Umap graph of (A). (C) Violin plots showing the expression of signature genes for different immune cell types. [file Image_1.tiff]

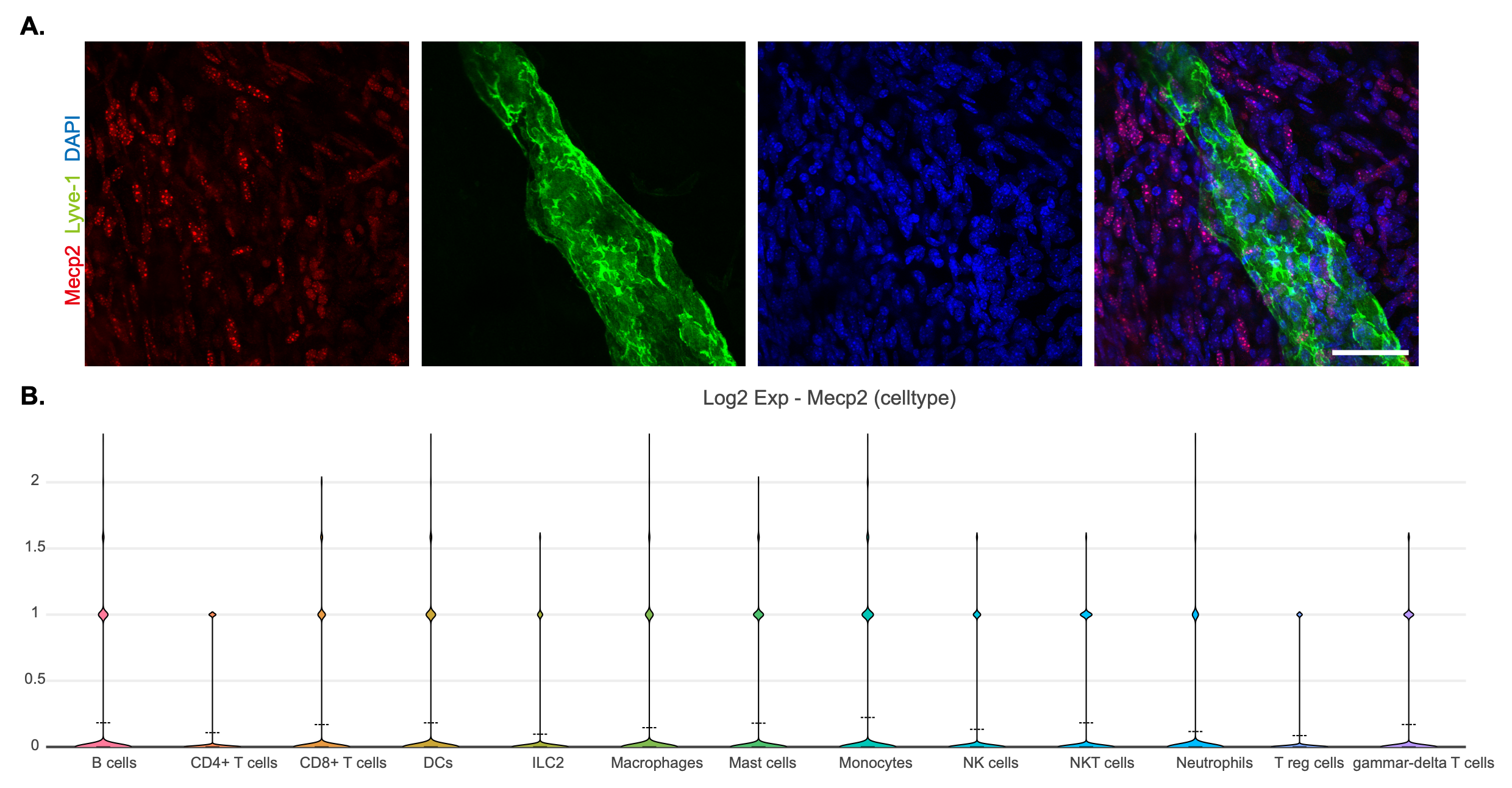

Supplement: Supplementary Figure 2 — Meningeal immune cells express Mecp2. (A) Representative confocal images of Mecp2 (red) and Lyve-1(green) staining in the dura mater of 1-month-old WT mice. Scale bars: 50μm. (B) Violin plots representing the Log2 expression of Mecp2 in different meningeal immune cells from WT mice. [file Image_2.tiff]
